# Advances in modelling the human microbiome–gut–brain axis *in vitro*

**DOI:** 10.1042/BST20200338

**Published:** 2021-02-05

**Authors:** Chrysanthi-Maria Moysidou, Róisín M. Owens

**Affiliations:** Department of Chemical Engineering and Biotechnology, University of Cambridge, Philippa Fawcett Drive, Cambridge CB3 0AS, U.K.

**Keywords:** 3D cell biology, gut-brain-axis, microbiome, organ-on-chip, organoid

## Abstract

The human gut microbiome has emerged as a key player in the bidirectional communication of the gut–brain axis, affecting various aspects of homeostasis and pathophysiology. Until recently, the majority of studies that seek to explore the mechanisms underlying the microbiome–gut–brain axis cross-talk, relied almost exclusively on animal models, and particularly gnotobiotic mice. Despite the great progress made with these models, various limitations, including ethical considerations and interspecies differences that limit the translatability of data to human systems, pushed researchers to seek for alternatives. Over the past decades, the field of *in vitro* modelling of tissues has experienced tremendous growth, thanks to advances in 3D cell biology, materials, science and bioengineering, pushing further the borders of our ability to more faithfully emulate the *in vivo* situation. The discovery of stem cells has offered a new source of cells, while their use in generating gastrointestinal and brain organoids, among other tissues, has enabled the development of novel 3D tissues that better mimic the native tissue structure and function, compared with traditional assays. In parallel, organs-on-chips technology and bioengineered tissues have emerged as highly promising alternatives to animal models for a wide range of applications. Here, we discuss how recent advances and trends in this area can be applied in host–microbe and host–pathogen interaction studies. In addition, we highlight paradigm shifts in engineering more robust human microbiome-gut-brain axis models and their potential to expand our understanding of this complex system and hence explore novel, microbiome-based therapeutic approaches.

## Introduction

**Figure 1.**
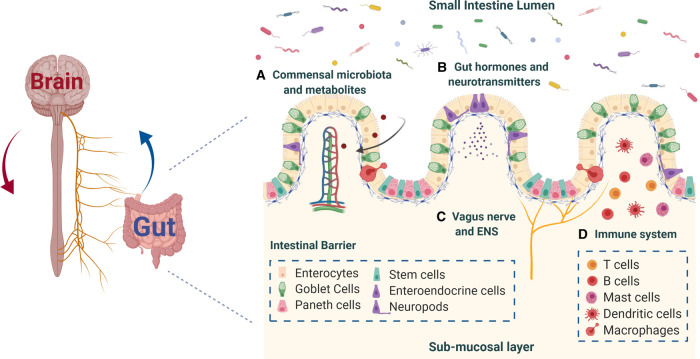
Communication pathways of the microbiota-gut-brain axis. The proposed mechanisms through which microbiota can signal the brain: (**A**) production of microbial metabolites that can reach the brain via the systemic circulation; (**B**) enteroendocrine system and gut hormone and neurotransmitter signalling pathways; (**C**) vagus nerve and ENS signalling pathways; (**D**) recruitment of the immune system, cytokine release and neuroimmune interactions. Created with BioRender.com.

Over the past decades, the integral role that gut microbiota play in human health and disease has been extensively studied and it is now apparent that the intestinal flora is a critical determinant and regulator of host physiology [1]. The gastrointestinal (GI) tract harbours a complex and dynamic population of over 100 trillion microbes, which have co-evolved with the host to form a mutually beneficial — symbiotic — relationship. These commensals are now known to participate in various fundamental processes of the human body, including digestion, energy metabolism, intestinal barrier function and homeostasis, immunity and production of vitamins and anti-inflammatory moieties [2]. The gut microbiota profile of each individual is unique and dynamic; established in early life and shaped with age by various factors (e.g., mode of birth, diet, exercise, genetics, lifestyle, the potential use of antibiotics and other medication) [2,3]. While microbiome maturation during development and ageing is naturally occurring and results in desirable compositional and functional changes with protective effects against inflammation and other disorders [1], a scrutiny of studies has revealed recently the connection between several pathophysiological conditions with an impaired gut microbiome, the effects of which extend beyond the gut and, in particular, to the brain [3–7]. Although the concept of bidirectional communication between the gut and the brain, termed the gut-brain axis, is far from new, it is now becoming apparent that microbes in the gut also participate in this interplay, considerably affecting neural function and pathophysiology (e.g., response to stress, susceptibility to autism, neurodegenerative diseases) [2,8]. Recent developments in the field suggest that communication in the microbiome-gut-brain axis occurs via several routes, (Figure 1) including the vagus nerve and the enteric nervous system (ENS), the immune system and enteroendocrine signalling pathways, as well as via release of microbial metabolites in systemic circulation [9]. However, the exact molecular and cellular mechanisms by which gut microbes transmit signals across the intestine and access the brain remain poorly understood not only due to the biological complexity of host–microbe interactions, but also due to the lack of appropriate tools [10,11].

Animal models and, specifically germ-free and gnotobiotic mice, have been invaluable for expanding our understanding on how microbiota and its alterations shape GI and brain (patho-)physiology and for establishing causal links between specific microbial cohorts and disease phenotypes [12–15]. But these models exhibit several limitations that render translation of data from animal to human systems quite challenging. Besides the ethical considerations, the high expenses, the time-consuming and labour-intensive nature of animal studies, rodents often fail to recapitulate human conditions due to inter-species differences related to gut topology, immune system and brain function (e.g., cognition), as well as differences in the wild-type microbiome profile and molecular mechanisms involved in the onset and progression of diseases [12,16]. While these models are still relevant and useful for a breadth of applications, in recent years, *in vitro* models of human systems have emerged as powerful tools that can be used in parallel with or, in some cases, alternative to animal studies. Currently, a wide range of engineered *in vitro* models are available, aiming to systematically investigate the complex cross-talk of microbes and human tissues in a more simplified context, within well-controlled and reproducible conditions for the evaluation of individual cell/tissue responses [11,12,16,17]. Early attempts involved conventional culture setups where intestinal cells were exposed to distinct microbiota, or neuronal cells were exposed to circulating gut-derived microbial metabolites, and the effects on host cells were evaluated via various assays, including permeability assays, multi-omic analyses, TEER, imaging [18–23]. Other approaches involved multi-compartment fermenting bioreactors, mimicking intestinal physiological characteristics along with the growth of commensal microbes, facilitating indirect, long-term studies [24–26]. Although such models allowed insight into many aspects of host-microbiome cross-talk, their inherent drawbacks (e.g., use of cell lines with cancerous origin, lack of bacterial and host cellular diversity, oversimplification, lack of host feedback mechanisms, absence of representative cross-talk) [12,17] restrict the extent to which the *in vivo* situation can be emulated to capture the complexity of the microbiome-gut-brain axis in its entirety. Therefore, novel bioengineering tools have been called for, for the development of more physiologically relevant human *in vitro* models. This minireview highlights the latest advances and paradigms in engineering such models, with the potential to generate more accurate and translatable data and, hence, (i) dissect the intricate interplay of microbiota and the gut-brain axis, (ii) improve our understanding of the microbiota alteration effects on host pathophysiology and (iii) lay the foundation for the development of new, personalised, microbiome-based drug and treatment approaches.

## Advanced tools for modelling the human microbiome-gut-brain axis *in vitro*

Over the past decades, a series of advances in three-dimensional (3D) cell biology and tissue engineering have enabled researchers to build more robust tools to better recapitulate native human tissue *in vitro* [27–29]. The discovery of induced pluripotent stem cells (iPSCs) and the subsequent establishment of long-term organotypic intestinal and brain cultures derived from human subjects enabled the development of novel cell systems, opening new avenues for expanding our scientific knowledge on host-microbiome interactions, among other applications [10,29]. In parallel, bioengineering approaches, that make use of biomimetic substrates (i.e., scaffolds and hydrogels) in combination with the appropriate cell sources and biochemical and biophysical cues, have gained a lot of attention recently for modelling tissues of higher biomimicry and physiological relevance [28,30]. In addition, new culturing technologies, using advanced media formulations and anaerobic conditions in specially designed platforms, have made possible mechanistic studies of difficult-to-culture or previously uncultivable gut microbes [31–33]. Such methods, along with powerful high-throughput next-generation genomic and metagenomic sequencing, permitted compositional and functional analyses to determine the dynamics of the complex gut microbiota community and their role in human health, completely transforming research approaches in the field [34,35].

### Organoids

**Figure 2.**
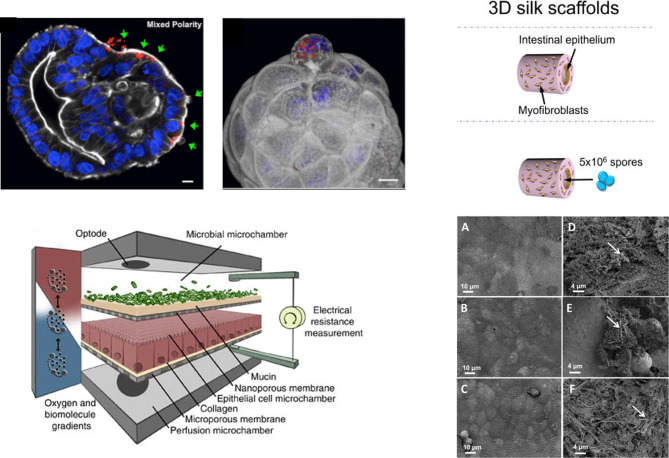
Examples of *in vitro* human intestinal models for studying host-microbe and host-microbiome interactions. (**A**) *S. Typhimurium*-mCherry infection of human enteroids. Selective invasion of the exposed apical surface (green arrows) of a mixed polarity enteroid (left). 3D confocal reconstruction of *S. Typhimurium*-mCherry within an epithelial cell in the process of extruding from the apical enteroid surface (right) (nuclei in blue, actin in white). Adapted from [45] under the Creative Commons license; (**B**) Conceptual diagram of the HuMiX model for the representative co-culture of human epithelial cells with gastrointestinal microbiota. Reproduced from [55], under the Creative Commons license; (**C**) Schematic of *C. difficile* infection in the 3D scaffold tissue model (left) and scanning electron microscopy of uninfected (left column) and infected with UK1 *C. difficile* 3D scaffolds (right column) at 4, 24 and 48 h (right). Adapted from [23].

Organoids have proven to be valuable *in vitro* cell systems for various biomedical applications, ranging from tissue homeostasis, disease modelling and drug testing to regenerative medicine and host–microbe interactions [36,37]. Currently, various protocols exist for establishing both GI and brain organoids, derived from adult tissue biopsies and iPSCs. These 3D self-organised tissue constructs exhibit *in vivo-*like architecture, regional specification and diverse cellular subtypes, more faithfully mimicking major features of the human native tissues compared with cell lines and animal models [10,38–41]. The presence of a variety of functional enteroendocrine cells in GI organoids, and in particular enterochromaffin cells which have been shown to trigger the ENS and to transduce chemosensory signals to the brain, allows modelling of aspects of the bidirectional communication of the gut-brain axis [42], while various GI organoid systems have offered valuable insight into both host-commensal and host–pathogen interactions. (See Table 1) As the lumen of GI organoids is enclosed in the centre of the construct and the basal membrane is displayed outwards, a popular technique to deliver microbiota or their metabolites to the apical surface of the epithelium is microinjection [43,44]. With this method, the internal niche required by microbes is preserved and is more suitable for studying long-term interactions with commensals or pathogens that normally infect the host from the lumen. However, it requires special equipment and it is quite challenging to perform reproducible and quantitative experiments, while damage of organoids during the process is often [44]. In an attempt to overcome these challenges, Co and collaborators [45] developed a technique to reverse the polarity of enteroids, to expose their apical surface to the media without compromising the structure and function of the intestinal constructs, and successfully used this to identify the infection patterns of invasive enteropathogens (Figure 2A). Alternatively, GI organoids are routinely grown before enzymatically dissociated and reseeded onto Transwell culture inserts or Matrigel/ECM coated dishes, where they form monolayers that comply with the epithelial barrier dynamics and allow for exposure of the apical/luminal surface to microbes or their metabolites, added in the culture medium [10,44]. Although these monolayers contain the same cellular diversity as the organoids they are derived from, they fail to capture the 3D microenvironment of the native tissue and may not be suitable for long-term experiments [10,44]. Finally, disruption of organoids into suspensions, and then mixing with microbes and subsequent cultivation in 3D matrices to reform organoids, has also been used in host–microbe interaction studies. Despite the straightforward nature of this method, such approaches do not accurately capture the infection mechanism for all types of microorganisms, as some may interact with the basal side of the cells within the constructs, inducing non-specific responses [44]. There is currently insufficient evidence of one technique being superior to the other in capturing the *in vivo* situation, rather, the choice of method is based on the nature of the question being asked [10]. Despite the great potential of GI organoids as *in vitro* human models, the inherent limitations of reproducing age-associated structural and functional aspects of the native tissue, the batch-to-batch heterogeneity in size and, most importantly, the lack of essential components of their living counterparts, including the vasculature, the ENS and the immune system, hinders their use in studies looking at microbiome-gut-brain signalling pathways. To overcome this challenge, efforts are being made to develop co-cultures of GI organoids with immune cells and/or enteric neurons [46,47]. Brain organoids have also been used to study neuro-immune, neuro-endocrine [48] and host–microbe interactions, including Zika virus, [49–51] *Toxoplasma gondii*, [52] congenital human cytomegalovirus (HCMV) [53] and Japanese encephalitis virus (JEV) [54]. Advances in (patient) iPSC-derived GI, ENS and brain organoids with vagal nerve neurons, as well as in methods to co-culture these components, could provide in the future a means to model the gut-brain connection and study the role of microbiota in various aspects of human health and disease [48].

**Table 1.** Examples of human GI organoid cultures for studying host–microbe and host–pathogen interactions.

Microbe(s)/metabolites	Organoid	Method	Main findings	References
*Clostridium difficile*	Proximal colon organoids	Microinjection	*C. difficile* reduces MUC2 production and not MUC1, but is not capable of altering host mucus oligosaccharide composition.	[56]
	iPSC derived intestinal organoids, small intestine	Microinjection	*C. difficile* persistence and toxin production disrupts the epithelial paracellular barrier function.	[57]
Enterohemorrhagic *Escherichia coli* (EHEC)	Proximal colon organoids	Dissociation –Transwell monolayers	MUC2 and protocadherin 24 (PCDH24) are targeted by EHEC at early stages of infection. EHEC reduces colonic mucus and affects the brush border cytoskeleton in the absence of commensal bacteria.	[58]
Enteroaggregative *E. coli* (EAEC)	Enteroids	Dissociation – human intestinal monolayers	Differences in the intestinal segments as well as in donors/hosts contribute to unique patters of adherence and infection.	[59]
Enterotoxigenic and Enteropathogenic *E. coli*	Enteroids, co-cultured with human macrophages	Dissociation –Transwell monolayers	Macrophages enhance barrier function and maturity of enteroid monolayers. Macrophage and enteroid-derived cell co-ordinated response to infections.	[46]
*E. coli* ECOR2 and K-12 MG1655	Organoids — immature intestinal epithelium	Microinjection	Microbial colonisation of HIOs leads to hypoxia driven responses, increased antimicrobial peptide production and maturation of the mucus layer, and improved barrier function.	[60]
*Salmonella Typhimurium*	iPSC derived intestinal organoids, small intestine	Microinjection	HIOs effectively model aspects of *S. Typhimurium*-intestinal epithelium interactions. *S. Typhimurium* stimulation alters the gene expression patterns in HIOs.	[61]
	iPSC derived intestinal organoids, small intestine	Dissociation –Transwell monolayers	*S. Typhimurium* targets human-specific pathways by inducing host transcriptional changes (cytoskeletal rearrangement, polarized cytokine release, and hampering host immune defense system).	[62]
	iPSC derived intestinal organoids, colon and ileum	Microinjection	The IL-22 pathway facilitates control of microbial infection of the human intestinal epithelium, involving enhanced phagolysosomal fusion.	[63]
	Apical-out vs basal-out enteroids	Infection via media	Bacteria can induce actin ruffles to invade the human IECs and preferentially invade apical surfaces.	[45]
*Listeria monocytogenes*	Apical-out vs basal-out enteroids	Infection via media	*L. monocytogenes* invades the human IECs via attachment to basal receptors.	[45]
Human noroviruses (HuNoVs)	iPSC derived and IBD patient derived intestinal organoids, small intestine	Dissociation monolayers	Bile is required for strain-dependent HuNoV replication. Lack of appropriate histoblood group antigen expression in intestinal cells restricts virus replication.	[64]
*Shiga* toxin (Stx) producing *E. coli* O157:H7 and commensal *E. Coli*	iPSC derived intestinal organoids	Microinjection	Commensal *E. coli* remained within the lumen and did not cause damage. O157:H7 induced loss of actin and epithelial integrity and increased reactive oxygen species production. Both commensal and O157:H7 up-regulated genes associated with gastrointestinal maturation. O157:H7 up-regulated inflammatory responses and resulted in recruitment of human neutrophils.	[65]
Enteroviruses (Echovirus 11 (E11), Enterovirus 71 (EV71)) and Coxsackievirus B (CVB),	Stem cell-derived organoids from the small intestine		Virus-specific activation of antiviral and inflammatory signalling pathways in response to infection. Enteroviruses infect specific cell populations in the human intestine.	[66]
*Helicobacter pylori*	Gastric organoids	Microinjection	*H. pylori* induces inflammatory response. IL8 expression was substantially higher in gland-type organoids than in pit-type organoids.	[67]
Butyrate	Foetal small intestinal organoids		Butyrate affects cytokine responses in epithelial cells and enhances maturation markers and RA production.	[68]
Indoleacrylic Acid (IA) produced by *Peptostreptococcus* Species	Colonoids		IA promotes intestinal epithelial barrier function and mitigates inflammatory responses. IBD patient microbiota show diminished capability to utilise mucins and metabolise tryptophan.	[69]

### Organs-on-chips

In parallel with advances in 3D cell biology and organotypic cultures, organs-on-chips (OOCs), alternatively called microphysiological systems (MPS), have also been used for modelling aspects of the microbiota-gut-brain axis. Combining principles of microengineering and fluidics with trends in growing cells in 3D, such models allow for cultivating human tissues in a more biomimetic microenvironment, where cells are exposed to tissue-relevant biochemical and biophysical cues (e.g., fluid shear stress, peristalsis) [70,71]. In addition, OOCs offer unparalleled, independent spatiotemporal tuning and control over multiple key factors of the cell system (e.g., O_2_, pH), *in situ*, automated monitoring and sample analysis along with downstream analysis, as well as the potential to study cell-cell and cell-niche interactions [71]. The benefits of these animal-free and more physiologically relevant models have been exploited recently for the development of guts-on-chips, aiming to mimic specific tissue structural and functional features (e.g., villus-crypt formation, mucus layer). Such approaches for engineering the gut microenvironment are superior to conventional *in vitro* models, as they enable continuous supply of nutrients and waste removal, incorporation of components of the vascular and immune systems and intestinal flora, [70,72] (See Table 2) and, in some cases, application of mechanical deformations to mimic gut peristaltic movements [73]. Some approaches are also focused on incorporating sensing units that allow for real-time monitoring of key parameters and factors, such as O_2_ [74] and barrier integrity, [75,76] which are important readouts for studies looking at the effects of microbes on the epithelium. Among the latest achievements in this field is the development of gut OOCs using hiPSCs and organoid-derived cells, both from healthy individuals or patients, in order to generate models that better capture the complexity of the intestinal epithelium in each condition [77]. Insights into host-microbiome interactions were achieved recently using this technology. For example, Shah et al. built a modular gut OOC that recapitulates the human GI-microbiome interface (Figure 2B) and used it to study the metabolic and immunological responses of the intestinal epithelium upon co-culture with commensal anaerobes [55]. A more recent approach modelled a human anaerobic intestine-on-chip, establishing a hypoxia gradient across the endothelium-epithelium interface, and was successfully used for an extended, stable co-culture of human intestinal tissue with a highly complex human-derived gut microbiota cohort, providing a valuable tool for more in-depth studies of host-microbiome cross-talk [74].

**Table 2.** Examples of gut-microbiome OOC devices

Cell source	Microbe(s)/metabolite(s)	Main findings	References
Caco-2BBE, Human Peripheral Blood Mononuclear cells, Human Microvascular Endothelial cells (HIMECs)	*Lactobacilus acidophilus, Lactobacilus plantarum, Lactobacilus paracasei, Lactobacillus delbrueckii subsp. bulgaricus, Bifidobacterium breve, Bifidobacterium longum, Bifidobacterium infantis, Streptococcus thermophiles*, LPS	Probiotic and antibiotic therapies can suppress villus injury induced by pathogenic bacteria. Lack of epithelial deformation triggers bacterial overgrowth similar to that observed in patients with ileus and IBD. Immune cells and LPS endotoxin together stimulate epithelial cells to produce proinflammatory cytokines.	[78]
Primary human colon epithelial cells isolated from patient-derived organoids interfaced with HIMECs	EHEC, soluble metabolites isolated from bioreactor cultures of complex populations of murine or human intestinal commensal microbes	Human microbiome metabolites increased EHEC's ability to induce epithelial damage, rather than the mouse microbiome products protecting against the damaging effects of this infectious pathogen.	[79]
Caco2 BBE, HIMECs, Ileal organoid-derived epithelial cells from healthy individuals and patients	*Bacilus fragilis* (9343), healthy human complex microbiota maintained stably in gnotobiotic mice and fresh gut microbiome from human infant stool samples	A physiologically relevant low-oxygen microenvironment sustains a diverse community of commensals with increased abundance of obligate anaerobes, resembling the *in vivo* situation. Co- culturing intestinal epithelium with either a single commensal or bacterial cohorts under physiologically relevant anaerobic conditions enhances epithelial barrier function compared with aerobic conditions.	[74]
Caco-2 CCD-18Co Primary CD4+T	*Lactobacillus rhamnosus GG (LGG), Bacteroides caccae*	Co-cultured microorganisms alter expression of miRNAs linked to colorectal cancer in Caco-2 cells. LGG induces the accumulation of GABA in epithelial cells.	[55]

Similarly, OOC technology has been used for generating more robust models of the BBB and/or the brain, [80–86] which have shown great potential for testing whether drug candidates can actually cross the BBB and reach their target in the brain [87]. However, such models are of great importance for modelling the BBB and brain *per se*, as current gold standards often fail to capture the complex structural and functional aspects of the human brain [81]. Although the effects of gut commensal metabolites on the brain have been demonstrated in *in vitro* models of the human BBB, [19] the potential of single BBB and/or brain OOCs has not yet been employed in microbiome research. Indeed, the benefits of each of the OOCs required to mimic the microbiota-gut-brain axis *in vitro* have been demonstrated individually [88]. However, there is much interest in functionally coupling individual OOCs via their endothelium/vascular channel in an *in vivo-*like sequence towards multi-organ OOC systems that reconstitute the role of vasculature perfusion and the cross-talk between the tissues of interest [89–91]. Recently, this concept was shown to be particularly apt for studying the effects of microbiota in the brain in a study coupling individual OOCs mimicking the gut-liver-kidney-brain axis, where the toxicity of the microbiome metabolites trimethylamine (TMA) and trimethylamine-N-oxide (TMAO) were tested and exhibited that TMAO can pass through the BBB to reach the NVU [91].

### 3D Bioengineering approaches

Even though the fast-growing OOC technology has emerged as a highly promising tool for various biomedical applications, the field is still relatively young, with various challenges to be addressed [88]. Indeed, OOC approaches have been shown to more faithfully mimic the *in vivo* microenvironment compared with conventional culture systems. However, in most cases they form a quasi-3D cell system, not entirely capturing structural and functional features of the native tissue. To overcome this limitation, TE approaches have been called for generating truly 3D tissues, where gels and scaffolds are used as templates, combined with the appropriate cell source (i.e., stem cells, organoids) and tissue-relevant environmental cues [92,93]. Such bioengineering approaches facilitated the development of more robust gut-like [94–100] and brain-like [101–105] tissue equivalents, significantly improving our ability to model various aspects of their (patho-) physiology. In one of the seminal studies towards this end, the researchers engineered 3D porous scaffolds using silk protein, featuring a hollow lumen with a polarised monolayer of human intestinal epithelial cells, supported by myofibroblasts cultivated in the scaffold bulk. These intestinal tissues exhibited characteristic functions of the human intestine, including mucus layer formation and low oxygen tension in the lumen, while able to interact with gut-colonising bacteria[106] as well as supporting studies of *C. difficile* germination, colonisation, toxin production and epithelial damage (Figure 2C) [23]. More recently, the group developed a perfused bioreactor system to host these intestinal tissues, offering better control over oxygen levels and physicochemical parameters, [107] while also reported the integration of a functional stem cell-derived ENS towards innervated intestinal constructs, particularly apt for studies looking at ENS-mediated communication networks [108].

**Figure 3.**
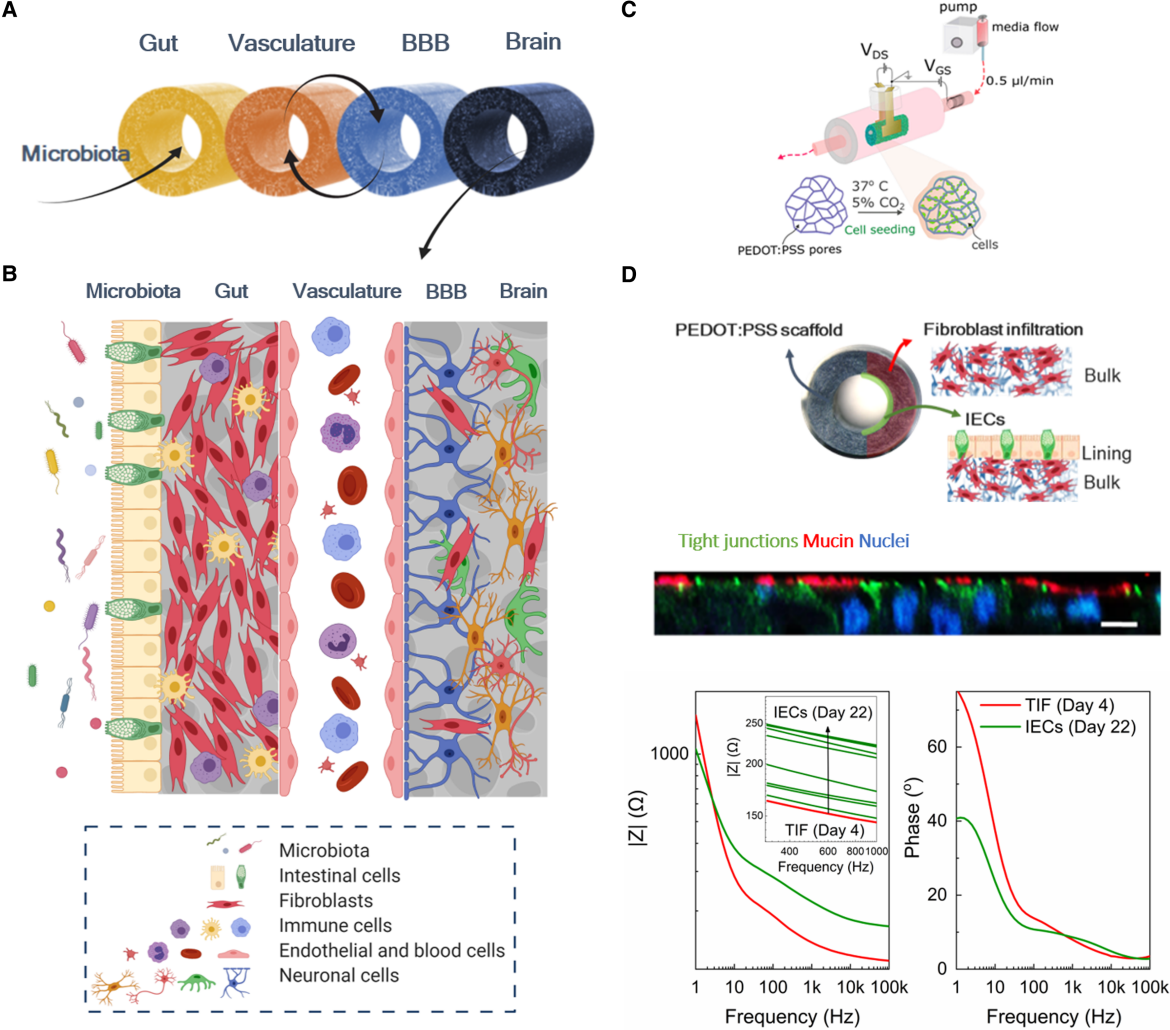
Design and tools of the IMBIBE platform. (**A**) Modules of the microbiota-gut-brain axis segments are built to operate independently but can also be interconnected to mimic the *in vivo* situation, by means of fluidic coupling and interconnection of each tissue equivalent via their bulk compartments; (**B**) Illustration of the biological components of the complete IMBIBE platform; Created with BioRender.com (**C**) Schematic of the structure and setup of the ‘Tubistor’, the novel 3D bioelectronic device for building each module of the IMBIBE platform. Reproduced from [109] under the Creative Commons License. (**D**) The gut module of the IMBIBE platform, hosted in the new generation of the device in (**C**) — the ‘L-Tubistor’ — modified to better capture the native tissue architecture. A schematic illustration of the intestinal model showing the organisation of different cell components in the hollow tubular electroactive scaffolds of the L-Tubistor (top). Snapshot of *z*-stacked confocal images illustrating the brush border of the polarised intestinal epithelial layer on the scaffold lumen lining (middle; scale bar 20 µm). Representative graph of electrical monitoring of the intestinal model showing the response of the electroactive scaffolds to tissue formation from day 4 of fibroblast culture to day 22 of intestinal cell culture (overall day 26; bottom). Adapted from [110] under the Creative Commons License.

However, in most cases 3D bioengineered tissues lack in-line monitoring units that would allow for real-time assessment and interrogation of the cell state and activity, mainly relying on end-point assays and downstream analysis [16]. To overcome this limitation, we recently developed 3D tubular scaffolds based on conducting polymers that act both as hosts of 3D cell systems and as active elements for continuous monitoring of cell activity and integrity. By tailoring the electrical, mechanical and biochemical properties of the materials we were able to generate a bioelectronic platform for successfully growing 3D mammalian tissue over a period of 4 days during which electrical readouts helped us to monitor cells and distinguish between adhesion and barrier tissue formation [109]. This novel *in vitro* tool forms the basic technological brick for our ERC funded project ‘IMBIBE’ (grant agreement No. 723951), which aims to generate a complete platform of the human microbiota-gut-brain axis with integrated monitoring and sensing capabilities (Figure 3A–C). Bringing together principles of materials science, tissue engineering, 3D cell biology and bioelectronics, we are currently building advanced models of the GI and the BBB/NVU, towards robust and more physiologically relevant human *in vitro* models. The innovation in IMBIBE comes from focusing on an *in vivo*-like 3D environment, using novel sources of human cells (i.e. cell lines, stem cell-derived or organoid-derived cells), while also benefitting from cutting edge organic electronic technology for multi-parameter and real-time monitoring, compatible with and adapted to the cell systems. Our models are designed to have sufficient complexity to produce predictive data, useful in understanding the interactions between the biological components, while being reductionist enough to facilitate alterations of system parameters to answer specific biological questions. In parallel, the integrated electronic assays allow for continuous collection of data to assess the model with a variety of different readouts, targeted for different biological questions. We recently demonstrated the unparalleled capabilities our systems bring in engineering tissues *in vitro* by generating the 3D bioelectronic human intestinal module of the IMBIBE platform, the L-Tubistor [110]. The module is based on the aforementioned tubular electroactive scaffolds, in the centre of which a hollow channel was introduced to mimic the native tissue luminal architecture. The L-Tubistor scaffolds were shown to support the growth and maintenance of an *in vivo-*like stratified and polarised intestinal tissue for the extended period of ∼1 month. Continuous monitoring of the changes in the electrical properties of the scaffold, induced by interactions with cells and extracellular matrix, provided unprecedented real-time information on tissue formation and integrity with a highly sensitive multi-modal operation (i.e., as an electrode and as a transistor), in a non-invasive manner (Figure 3D). The methodology for tissue growth within the L-Tubistor can be adapted to build the other modules of the IMBIBE platform, while its compatibility with fluidics will enable the functional coupling of these modules towards the desired multi-organ-on-a-chip. We envision that our IMBIBE platform will aid the research efforts to (i) elucidate the role of microbiota in the gut-brain axis communication, (ii) to study how diet and impaired microbiota profiles affect various (patho-) physiologies, and (iii) to test personalised medicine approaches for disease modelling and drug testing studies.

## Conclusions

The intricate interplay between the gut and the brain has long been appreciated. However, over the past decades, great attention has been given to the role of microbiota in this bidirectional communication and its effects on various aspects of health and disease. Although much of our understanding about the microbiota-gut-brain axis comes from animal models, their inherent shortcomings and inability to reproduce the human situation have turned the attention of the research community to *in vitro* models. The advent of hiPSCs and organoids provided researchers with robust organotypic cell systems for emulating parts of the gut-brain axis and interfacing them with microbiota. In parallel, OOCs and 3D bioengineered tissues have emerged as powerful tools, advancing our ability to more faithfully emulate human tissues *in vitro* within a controllable and reproducible environment. Despite their reductionist nature, various gut-microbiome models have successfully been implemented in studies looking at host–microbe interactions, offering crucial insight into the effects of microbes on the intestinal epithelium homeostasis and infection mechanisms. However, there is currently no *in vitro* model of the complete microbiota-gut-brain axis and many challenges remain to be addressed before such a single platform is engineered (e.g., inclusion of all representative cell types in stable and viable co-cultures for long-term experiments, *in situ* sample analysis and monitoring units). Functional, modular coupling of the microbiota-gut-brain axis experimental constituents holds great potential for effectively modelling the axis in its entirety. It is apparent that this is a multidisciplinary field, requiring close collaboration of different fields to resolve both technological and biological obstacles. We expect that further advances in bioengineering, stem cell biology and organoid systems will facilitate the generation of robust and reliable *in vitro* models to support comprehensive studies of the mechanisms underlying the complex cross-talk in the microbiome-gut-brain axis, as well as to explore novel microbiome-based therapeutic approaches.

**Box 1. Glossary**

*Microbiota and microbiome*: The terms are often used interchangeably to describe the community of commensal, symbiotic and pathogenic microorganisms present in a defined environment, including the body or parts of the body, such as the gut. See [111] for a detailed description of microbiome definitions. The term microbiome is mostly used to refer to the collective genomes of the microorganisms in a specific environment, while the term microbiota refers to the assemblage of the microorganisms *per se* [112].

*Gnotobiotic animals:* Refers to animals in which every microorganism present is defined. Germ-free mice, commonly used in microbiome research, are one class of gnotobiotic animals, as well as mice associated with defined bacterial communities [113].

*Microbiota-Gut-Brain axis:* The bidirectional communication between the microbiota, the gut and the brain through encompassing distinct pathways of the autonomic nervous system (ANS), the enteric nervous system (ENS), the hypothalamic-pituitary-adrenal axis (HPA), the neuroimmune system and metabolites translocating from the intestinal mucosa into the bloodstream. See [1] for a comprehensive review.

*Organoids*: Generally the term is used to refer to self-organising *in vitro* structures resembling an organ. More specifically though ‘a genuine organoid should satisfy several criteria: (1) a 3D structure containing cells that establish or retain the identity of the organ being modelled; (2) the presence of multiple cell types, as in the organ itself; (3) the tissue exhibits some aspect of the specialised function of the organ; and (4) self-organisation according to the same intrinsic organising principles as in the organ itself’ [114]. Enteroids (small intestine) and colonoids (large intestine/colon) are precursors of organoids, formed at initial stages of the organoid cultures, containing only epithelial cell types. See [115].

*Organotypic cultures*: Approaches of reconstituting organ function *ex vivo,* including 3D cell culture systems, explants-tissue slices and organoids [116].

*Organs-on-chips (OOCs)*: Emerging interdisciplinary technology that combines principles of microfluidics and microengineering, cell biology, tissue engineering, aiming to develop miniature tissues and organs *in vitro* that capture key architectural and functional aspects of a specific human tissue. These models represent promising alternatives to animal studies for investigating physiological and pathological events of the tissue as well as for testing drug and treatment candidates by generating more accurate and translatable results. See [71,72] for latest advances and trends in the field.

## Perspectives

- The gut microbiome has emerged as a key determinant and regulator of gut-brain homeostasis. Although accumulating evidence links the intestinal flora with various diseases and disorders, the mechanisms underlying the intricate host-microbiome cross-talk have not yet been fully understood.
- Interspecies differences, among other shortcomings of animal models, have pushed researchers to look for more relevant human models. Several frameworks have been put forward to engineer *in vitro* models of the human microbiota-gut-brain axis, including organoid cultures, OOCs and 3D bioengineered tissues.
- Currently, no single platform exists recapitulating the complete axis. Efforts are focused on resolving both the biological and technological limitations of the current state-of-the-art, by generating more robust cell culture systems and by optimising the design, material properties and fabrication methods of the platforms that will support the maintenance and characterisation of the models, respectively.

## Open Access

Open access for this article was enabled by the participation of University of Cambridge in an all-inclusive *Read & Publish* pilot with Portland Press and the Biochemical Society under a transformative agreement with JISC.

## Abbreviations

(h)iPSCs: (human) induced pluripotent stem cells
3D: three-dimensional
BBB: blood-brain-barrier
ENS: enteric nervous system
GI: gastrointestinal
MPS: microphysiological systems
NVU: neurovascular unit
OOC: organs-on-chips
TE: tissue engineering
TEER: trans-epithelial/endothelial electrical resistance
